# Interventions to prevent iatrogenic anemia: a Laboratory Medicine Best Practices systematic review

**DOI:** 10.1186/s13054-019-2511-9

**Published:** 2019-08-09

**Authors:** Nedra S. Whitehead, Laurina O. Williams, Sreelatha Meleth, Sara M. Kennedy, Nneka Ubaka-Blackmoore, Sharon M. Geaghan, James H. Nichols, Patrick Carroll, Michael T. McEvoy, Julie Gayken, Dennis J. Ernst, Christine Litwin, Paul Epner, Jennifer Taylor, Mark L. Graber

**Affiliations:** 1 RTI International, Research Triangle Park, NC USA; 2 Division of Laboratory Systems, Center for Surveillance, Epidemiology, and Laboratory Services, Centers for Disease Control and Prevention, 1600 Clifton Road, NE, MS G25, Atlanta, GA 30333 USA; 3 Department of Pathology, Pediatrics Division, Stanford University School of Medicine, Stanford, CA USA; 4 Vanderbilt University School of Medicine, Nashville, TN USA; 5 Division of Neonatology, Intermountain Healthcare , St. George, UT USA; 6 Albany Medical Center, Albany, NY USA; 7 Julie Gayken Laboratory Consulting, St. Cloud, MN USA; 8 Center for Phlebotomy Education, Inc, Corydon, IN USA; 9 Clinical Immunology and Referral Testing, Medical University of South Carolina, Columbia, SC USA; 10 Paul Epner, LLC, Evanston, IL USA

**Keywords:** Iatrogenic diseases, Anemia, Phlebotomy, Clinical laboratory techniques, Blood conservation strategy

## Abstract

**Background:**

As many as 90% of patients develop anemia by their third day in an intensive care unit (ICU). We evaluated the efficacy of interventions to reduce phlebotomy-related blood loss on the volume of blood lost, hemoglobin levels, transfusions, and incidence of anemia.

**Methods:**

We conducted a systematic review and meta-analysis using the Laboratory Medicine Best Practices (LMBP) systematic review methods for rating study quality and assessing the body of evidence. Searches of PubMed, Embase, Cochrane, Web of Science, PsychINFO, and CINAHL identified 2564 published references. We included studies of the impact of interventions to reduce phlebotomy-related blood loss on blood loss, hemoglobin levels, transfusions, or anemia among hospital inpatients. We excluded studies not published in English and studies that did not have a comparison group, did not report an outcome of interest, or were rated as poor quality. Twenty-one studies met these criteria. We conducted a meta-analysis if > 2 homogenous studies reported sufficient information for analysis.

**Results:**

We found moderate, consistent evidence that devices that return blood from flushing venous or arterial lines to the patient reduced blood loss by approximately 25% in both neonatal ICU (NICU) and adult ICU patients [pooled estimate in adults, 24.7 (95% CI = 12.1–37.3)]. Bundled interventions that included blood conservation devices appeared to reduce blood loss by at least 25% (suggestive evidence). The evidence was insufficient to determine if these devices reduced hemoglobin decline or risk of anemia. The evidence suggested that small volume tubes reduced the risk of anemia, but was insufficient to determine if they affected the volume of blood loss or the rate of hemoglobin decline.

**Conclusions:**

Moderate, consistent evidence indicated that devices that return blood from testing or flushing lines to the patient reduce the volume of blood loss by approximately 25% among ICU patients. The results of this systematic review support the use of blood conservation systems with arterial or venous catheters to eliminate blood waste when drawing blood for testing. The evidence was insufficient to conclude the devices impacted hemoglobin levels or transfusion rates. The use of small volume tubes may reduce the risk of anemia.

**Electronic supplementary material:**

The online version of this article (10.1186/s13054-019-2511-9) contains supplementary material, which is available to authorized users.

## Background

Iatrogenic anemia, the development of anemia due to medical procedures, is a universal concern among critically ill patients. Adult intensive care unit (ICU) patients lose approximately 340–660 mL of blood per week to diagnostic testing [1, 2], with an 18% increase in the risk of anemia for each 50 mL of blood lost [3]. Over 70% of adult ICU patients are anemic by the second day of admission, and almost half will ultimately be transfused [4].

Much of the blood drawn for laboratory testing is discarded. Sanchez-Giron et al. [5] found that when standard volume tubes were used 91% (4612 mL) of blood remained after testing was complete, compared to 74% (1267 mL) remaining when small volume tubes were used. A recent cohort study of small volume tubes [6] found that they reduced the total volume of blood drawn per patient per day, but samples with fibrin present and total laboratory errors increased significantly (fibrin 0.3%, *p* < 0.001; total errors, 0.4%, *p* = 0.03).

Some researchers have questioned whether blood loss from diagnostic testing contributes significantly to inpatients’ development of anemia. They suggest instead that patients with severe illness have impaired erythropoiesis, which causes anemia and requires more diagnostic testing to monitor their illness [7]. Mathematical modeling suggests that it would take 40–70 days of 53 mL/day of blood loss for adults with normal body weight, hemoglobin at admission to the ICU at the midrange of normal, and active erythropoiesis to become anemic. However, the same models indicate that the hemoglobin concentrations of patients with reduced erythropoiesis, initial hemoglobin concentrations at the lower limit of normal, and low body weight, characteristics typical of ICU patients, who are exposed to increased phlebotomy may decline to 70 g/L or less by 9–14 days [8]. Average blood loss to diagnostic testing among adult ICU patients in one study was 77.8 mL/day [9].

Phlebotomy-related blood loss is even more profound and consequential in neonatal ICU patients: these infants lose 10 to 90% of circulating blood volume to phlebotomy in the first 2 weeks of life alone [10, 11]. Many ICU patients require transfusion, increasing the risks of infection, vascular overload, lung injury, sensitization, and transfusion reaction [12, 13]. Drugs that stimulate erythropoiesis and stringent transfusion guidance can reduce exposure to transfusions but not risks from anemia itself; the best strategy is to prevent the phlebotomy-driven anemia from the start [11, 14, 15].

Interventions to minimize phlebotomy blood loss include non-invasive testing, blood conservation devices and techniques, point of care testing (which requires less sample volume), and education or decision support tools to guide testing decisions [13, 16–22]. The uptake of these interventions varies among healthcare systems. Although individual studies have examined their efficacy, we found no previous synthesis of the evidence regarding these interventions. We conducted a systematic review of the efficacy of interventions to reduce phlebotomy-related blood loss and prevent iatrogenic anemia.

## Methods

We applied the first four steps of the LMBP systematic review method (Ask, Acquire, Appraise, and Analyze) to conduct this review and to evaluate the effectiveness of interventions that reduce blood loss [23]. A panel of experts (Additional file 1) in clinical care, laboratory medicine, systematic review, informatics, and patient safety identified relevant articles and provided individual input on the search strategy, review protocol (Additional file 2), and the interpretation of findings. The review assessed the following research questions regarding interventions to minimize phlebotomy-related blood loss and iatrogenic anemia: Does the intervention

- Reduce the volume of blood drawn?
- Reduce the decline in hemoglobin levels during admission, the incidence of iatrogenic anemia, or the need for transfusion?
- Lead to inadequate blood for testing, a need for additional blood draws, or patients not receiving appropriate testing, resulting in compromised care?

**Fig. 1:**
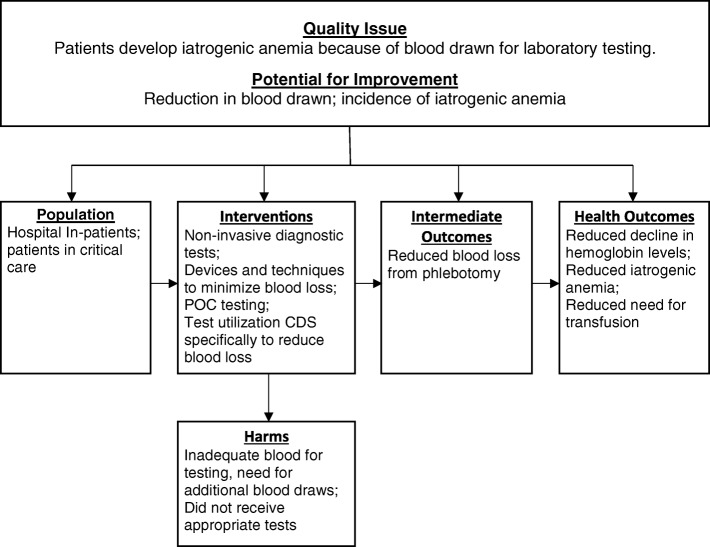
Analytic framework for Laboratory Medicine Best Practice systematic review of interventions to reduce or prevent the incidence of iatrogenic anemia

The analytic framework for the review is shown in Fig. 1.

Hospital inpatients included patients with an overnight stay who were not formally admitted. We expected studies of the interventions shown in the analytic framework, but included any identified intervention to reduce phlebotomy-related blood loss. Valid comparisons included intervention group to those among patients who did not receive the intervention or who were treated prior to intervention. For the volume of blood lost, we included studies published in English between January 1, 1990, and April 10, 2017; for other outcomes, we limited inclusion to studies published after January 1, 2000, to avoid potential bias from changes in transfusion policies.

A professional librarian searched PubMed, Embase, Cochrane, Web of Science, PsychINFO, and CINAHL for relevant citations using a tailored search strategy (Additional file 2). Relevant studies were also identified by expert panel members and by manual searches of bibliographies of relevant studies. We sought unpublished studies through expert panelists and relevant professional organizations but did not identify any. We excluded articles that (1) were letters, editorials, commentaries, or abstracts; (2) were set in a clinic, emergency room, or other outpatient facility; (3) sampled outpatient populations or health care providers; (4) were about test management or transfusion strategies not aimed at reducing blood loss; (5) did not include data on the health outcomes of the intervention; (6) did not have a valid comparator; (7) did not assess an outcome of interest; or (8) did not have an appropriate study design (e.g., case reports, case series, historical controls that were more than 2 years older than the intervention).

Two independent reviewers evaluated each retrieved citation for eligibility for inclusion. A team member abstracted data on study characteristics, interventions, outcomes, and results; a senior scientist reviewed each abstraction. Two senior reviewers independently appraised the quality of the studies using the A-6 scoring scale. Discrepancies were resolved by discussion and adjudication by the principal investigator if needed. Studies that scored 4 or less out of 10 were excluded from the analysis.

### Evidence synthesis and meta-analysis

We converted the volume of blood loss to milliliters per patient per day when possible, and decline in hemoglobin to grams per liter per day. Measures reported for the entire length of stay were converted to daily values by dividing the total by the average length of stay, and group totals were converted to patient-level measures by dividing by the total by the number of patients in the group.

Effect size rating was determined a priori based on clinical significance as minimal, 0 to < 10%, moderate, 10 to 30%, and substantial, more than 30%, for all outcomes except the decline in hemoglobin. For the decline in hemoglobin, a relative effect greater than 20% was considered substantial. We synthesized evidence by intervention type and outcome. We rated the body of evidence as high, moderate, suggestive, or insufficient based on the number of studies, the study ratings, and the magnitude of the effect size (Additional file 3) and as described by Christenson et al. [23]. In brief, a high level of evidence requires three or more good quality studies with substantial effects; moderate evidence requires two good quality studies with substantial effects or at least three good quality studies with moderate effects; and suggestive evidence requires one good study with substantial effect, two good studies with moderate effects, or at least three fair studies with moderate effects. There must be at least moderate evidence to support the effectiveness of a laboratory practice. (Insufficient evidence does not rule out the potential value of the practice. Frequently, it indicates a need for additional evidence assessing the effect of the practice.) We also considered the consistency of the effect across studies in rating the strength of the evidence.

Meta-analytic estimates were calculated for outcomes for which we had evidence from at least three independent studies with the same type of intervention and outcome. We used the methods of Hedges and Vevea [24]. Fixed effects modeling was used if the studies were homogeneous and random effects modeling if they were heterogeneous, based on the *I*^2^ test for heterogeneity.

## Results

### Search

**Fig. 2:**
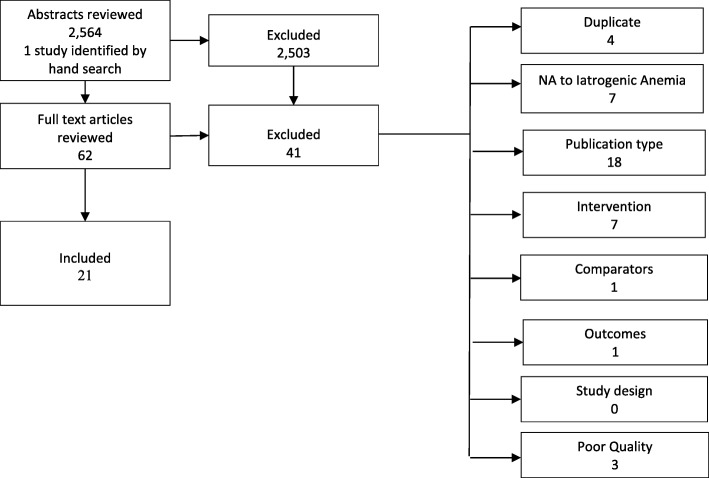
Literature search results

We retrieved 2564 abstracts from the database search and identified one study from hand searches of bibliographies. Twenty-four studies were included after the full-text review, but three studies were excluded because of poor study quality, leaving 21 studies for analysis (Fig. 2). The characteristics of the studies are included in the supplemental material (Additional file 4).

### Interventions reviewed

Five types of interventions were evaluated by the 21 papers: (1) small volume tubes, (2) closed blood sampling devices, (3) point of care testing, (4) staff guidance (education; institutional policies), and (5) bundled interventions that variously combined two more interventions. Details of the evidence on the interventions are listed in the supplemental material (Additional file 5).

### Impact of small volume or pediatric tubes

Three studies [5, 25, 26] investigated the impact of routine use of small volume or pediatric tubes among adults using pre-post study designs. Two studies, Dolman [25], graded as good quality, and Sanchez-Giron [5], graded as fair quality, reported reduction in blood loss. Dolman et al. [25] reported that small volume tubes reduced blood loss by 9.2 mL/day/patient (95% CI 5.1, 13.3), while Sanchez-Giron [5] reported a 73% reduction in median blood loss over 2 weeks of 9.8 mL/patient or 0.7 (95% CI 0.6, 0.8) mL/patient per day. They noted that small volume tubes provided sufficient blood for the most commonly requested tests. The size of the tubes differed between the two studies. In the study by Dolman et al. [25], the tubes used in the control group were 8.5 mL, 6.0 mL, and 2.7 mL, and the small volume tubes were 5.0 mL, 2.0 mL, and 1.8 mL. The control tubes used by Sanchez-Giron [5] were 4.9 mL, 2.7 mL, and 3.0 mL, and the small volume tubes were 1.1 mL, 1.2 mL, and 1.4 mL.

Kurniali et al. [26], graded as good quality, found that hemoglobin concentrations decreased 1.6 g/L less, after adjusting for length of stay, when small volume tubes were in use. Dolman et al. [25] reported that the cumulative risk of severe anemia (Hgb < 7.0 g/dL) was reduced by more than half (10% vs. 22%, *p* = 0.01) and the mean units of packed red blood cells transfused per patient decreased 27%, 1.6 units (95% CI -0.0, 3.2) after the change to small volume tubes.

In summary, the evidence is suggestive that small volume tubes mitigate the development of anemia, but insufficient to evaluate the effect on blood loss or the decline in hemoglobin levels.

### Closed blood sampling devices

**Fig. 3:**
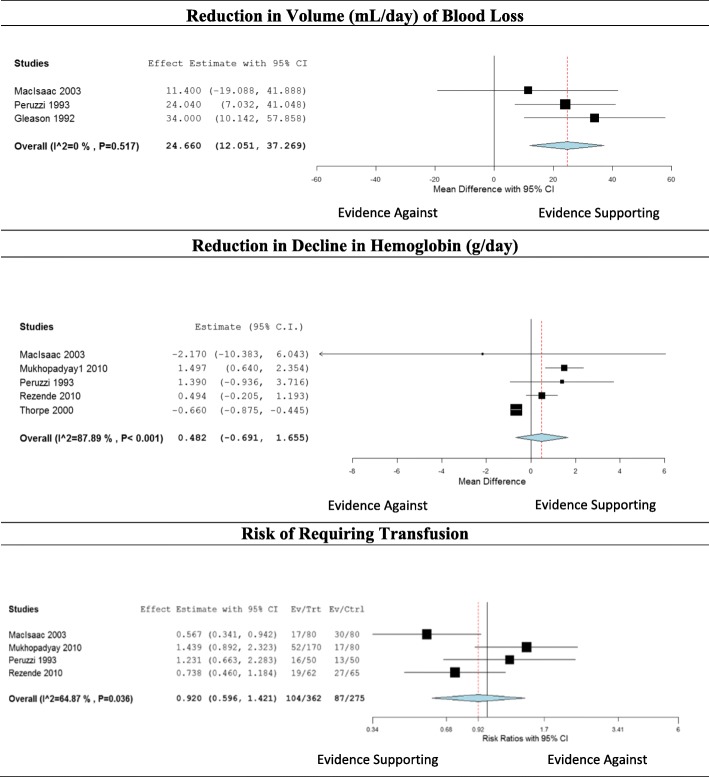
The impact of closed blood sampling devices

Eight studies [10, 27–33] examined the impact of using a closed blood sampling device (Table 1, Fig. 3). The studies by Mukhopadhyay [28, 29] and Gleason [31] were rated as fair quality; the other five studies were rated as good quality. The 2011 paper by Mukhopadhyay et al. [28] is a subgroup analysis of their 2010 study [29]; it includes only patients whose hemoglobin at admission was 115 g/L or higher and who were not transfused during the study. Widness et al. [10] studied a closed blood sampling device used in a pediatric population. They conducted a randomized controlled trial of an in-line blood gas monitor sampling device in the NICU of two institutions. The device returned blood to the infant, combining features of a closed blood sampling system and a point of care testing device, and was classified as a closed blood sampling device for analysis.

**Table 1:** The impact of closed blood sampling systems on blood loss, hemoglobin levels, and transfusion rates

Study (year)	Absolute effect (95% CI)	Relative effect	Effect rating	Quality rating	Consistency	Strength of evidence
Impact on blood loss (mL/day)						
MacIsaac (2003) [22]	11.4^a^ (-19.1, 41.9)	27%	Moderate	Good	Consistent	Moderate
Gleason (1992) [26]	34.0 (10.1, 57.9)	49%	Substantial	Fair		
Peruzzi (1993) [25]	24.0 (7.0, 41.0)	27%	Moderate	Good		
Widness (2005) [5]	NR^b^	24%	Moderate	Good		
Impact on hemoglobin decline (g/L/day)						
MacIsaac (2003) [22]	-2.2 (-10.4, 6.0)^c^	-1.2	Minimal	Good	Inconsistent	Insufficient
Mukhopadhyay (2010) [24]	1.5 (0.6, 2.4)	32	Substantial	Fair		
Mukhopadhyay (2011) [23]	0.3 (0.1, 0.5)^d^	6	Minimal	Fair		
Peruzzi [25]	1.4 (-0.9, 3.7)	36	Substantial	Good		
Rezende [27]	0.7 (-0.5, 1.2)	50	Substantial	Good		
Thorpe [28]	-0.7 (-0.9, -0.4)^c^	-1	Minimal	Good		
Transfusion risk (≥ 1 transfusion/admission)						
MacIsaac [22]	0.6 (0.3, 0.9)	75%	Substantial	Good	Inconsistent	Insufficient
Mukhopadhyay [24]	1.4 (0.9, 2.3)	-44%	Substantial	Fair		
Peruzzi [25]	1.2 (0.7, 2.3)	-17%	Moderate	Good		
Rezende [27]	0.7 (0.5, 1.2)	75%	Substantial	Good		

^a^Adjusted for median length of stay, which differed between groups

^b^Graphical representation only

^c^Greater decline among the intervention group

^d^Non-transfused patients only

Four studies [10, 27, 30, 31], all randomized controlled clinical trials, reported on the impact of closed blood sampling devices on the volume of patient blood loss (Table 1). In all four studies, patients in the intervention group lost less blood than patients in the control group. Three studies [10, 27, 30] found that closed blood sampling devices reduced blood loss by approximately 25%. The absolute reduction among adult patients ranged from 11.4 mL/day (95% CI -19.1, 41.9) [27] to 34 mL/day (95% CI 10.1, 57.9) [31]. Widness et al. [10] reported that the in-line blood gas monitor reduced total blood loss during the first 2 weeks of NICU admission by 24%. We included only the studies of adults in the meta-analysis due to differences between adults and newborns in total body blood volume and the volume of blood drawn for testing. The pooled estimate of blood conserved by the use of closed blood sampling devices in adult patients is 24.7 mL/patient/day (95% CI 12.1, 37.1).

Six studies [27–30, 32, 33] reported the impact of closed blood sampling devices on changes in patients’ hemoglobin levels during admission (Table 1). In four studies [28–30, 32], patients with closed blood sampling devices better maintained their hemoglobin levels than patients managed with conventional phlebotomy. The absolute improvement in hemoglobin decline ranged from 0.3 g/L/day [28] among untransfused patients with normal hemoglobin at admission to 1.5 g/L/day among all enrolled adult ICU patients [29], and the relative improvement ranged from 6% [28] to 50% [32]. In the other two studies [27, 33], patients on closed blood sampling devices had steeper declines in their hemoglobin levels than those on conventional phlebotomy. The median difference in the decline in hemoglobin was -2.2 g/L/day (CI not reported) [27] and -0.7 g/L/day (95% CI -0.9, -0.4) [33], a relative difference of -162% and -100%, respectively. Mukhopadhyay [28] was not included in the meta-analysis because it was a sub-analysis of their earlier study [29]. The pooled estimate was 0.5 g/L/day (95% CI -0.7, 1.7), indicating a slightly smaller decline among patients on closed blood sampling devices (Fig. 3). Given the inconsistency between studies, the evidence was judged insufficient to determine if closed blood sampling devices improved hemoglobin maintenance among adult ICU patients.

Four studies [27, 29, 30, 32] examined the impact of closed blood sampling devices on transfusion rates among adult ICU patients, with inconsistent results. Patients on closed blood sampling devices were less likely to have transfusions (two studies), with relative risks of 0.6 (95% CI 0.3, 0.9) [27] and 0.7 (95% CI 0.5, 1.2) [32]. In the other two studies [29, 30], patients in the intervention group were more likely than controls to have transfusions (RR 1.4, 95% CI 0.9, 2.3 [29], and 1.2, 95% CI 0.7, 2.3 [30]). The pooled effect estimate across the four studies was RR 0.9 (95% CI 0.6, 1.4) (Fig. 3).

In summary, there is moderate strength of evidence, consistent in effect, that closed blood sampling devices reduce blood loss due to diagnostic testing. The use of such devices reduces blood loss by about 25% compared to patients with conventional arterial pressure monitoring systems. Therefore, the findings of this systematic review support the use of blood conservation systems with arterial or venous catheters to eliminate blood waste when drawing blood for testing. The evidence is not sufficient to determine the impact of these devices on the decline in hemoglobin during ICU admission or the need for a transfusion.

### Point of care testing

Three studies [10, 34, 35], all graded as fair quality, examined the impact of point of care testing without closed blood sampling on blood loss and transfusion rates. Madan [22] and Mahieu [34] conducted retrospective medical record abstraction for newborns admitted to their NICUs. Salem et al. [35] conducted a prospective study of the volume of blood required by point of care testing compared to conventional laboratory testing among an adult ICU population. They found blood drawn for specific tests decreased overall, but they did not provide data on patient-level outcomes.

Cumulative cohort blood loss from electrolyte and bilirubin testing decreased by 19% (blood saved, 673 mL/pt. and 966 mL/pt., respectively) after the introduction of a point of care instrument [34]. In one study [22], transfusions within the first 14 days of admission decreased 46% (2.6 transfusions) after implementation of point of care testing. A second study [34] found point of care testing reduced transfusions only among very low birthweight (< 1500 g) infants: the number of infants that required a transfusion decreased by 22% after implementation, from 50 to 38.9%, and the mean number of transfusions per infant decreased from 2.5 to 1.6. The evidence was insufficient to determine the impact of point of care testing on any outcome.

### Bundled interventions

Four studies [36–39], all rated as fair quality, examined the impact of implementing multiple interventions at once (bundled interventions). The study population was adult ICU patients for three studies [36, 38, 39] and children with parapneumonic effusion for the fourth study [37]. The intervention bundles observed in the evidence base were:

- Small volume tubes, a closed blood sampling device, and decision tools, such as flow charts providing the amount of blood to draw for various tests [36];
- Small volume tubes, a closed blood sampling device, and non-invasive testing methods [39];
- Small volume tubes and a closed blood sampling device [38]; and
- A policy to minimize phlebotomy, microsample blood collection tubes, and reinfusion of blood drawn prior to obtaining a sample [37].

The three studies [36, 38, 39] conducted in adult ICUs found that blood loss among patients in the intervention group was at least 65% less than that among control patients. The absolute reduction ranged from 10.1 mL/day (95% CI 6.7, 13.2) [38] to 29.5 ml./day (CI not reported) [36]. The pooled estimate of blood saved by the interventions was 22.7 mL/day (95% CI 10.3, 35.1). Hassan et al. [37] found that their intervention saved 0.06 mL of blood per kilogram of patient weight per day (95% CI -0.06, 0.18) in a pediatric cohort.

The interventions had inconsistent effects on decline in hemoglobin [36, 39]. The intervention tested by Harber et al. [36] improved hemoglobin decline by 35% (2.3 g/L/day, 95% CI -1.4, 6.1), but the intervention tested by Riessen et al. [39] worsened hemoglobin decline slightly (-0.1 g/L/day (95% CI -0.5, 0.3; relative decline, 0.04%). Two studies [36, 39] reported that their interventions reduced the need for transfusion, by 44% (RR 0.7, 95% CI 0.1, 3.8) [36] and 400% (RR 0.3, 95% CI 0.1, 0.5) [39].

These studies provide suggestive strength of evidence that bundled interventions reduced the volume of blood loss among adult ICU patients by approximately 70%. The evidence on other outcomes was insufficient to determine the impact.

### Educational/policy interventions

Two studies, Foulke [40], rated as fair, and Matinez-Balzano [41], rated as good quality, reported on the impact of policy changes or educational interventions. The policy evaluated by Foulke et al. [40] required that less blood be drawn for laboratory tests, that small volume phlebotomy tubes be used, and that the total daily blood volume drawn be recorded on the patient’s chart. Martinez-Balzano [41] evaluated the effect of institutional guidelines on the appropriate ordering of arterial blood gas tests. Foulke et al. found that after implementation, test requisitions declined (9.3 ± 0.6 pre-intervention vs. 7.8 ± 0.5 after the intervention, *p* < 0.05); blood loss was reduced by 33% (43.6 ± 3 mL/day vs. 36.8 ± 3, *p* < 0.001), and the percentage of patients who had at least one transfusion was reduced from 10% (8 of 81) to 1.4% (1 of 70) (*p* < 0.05). Martinez-Balzano [41] found that blood gas requisitions decreased after the new guidelines by 41.5% (821.5 ± 257.4/month, *p* < 0.001); test requisitions for other commonly ordered tests did not change. The interventions differed greatly, and the evidence was insufficient to determine the impact of either intervention on any outcome.

### Summary of evidence

The strength of the evidence for each intervention and outcome is summarized in Table 2.

**Table 2:** Strength of evidence for each intervention-outcome pair

	Outcome (number of studies; strength of evidence rating)				
Intervention	Test requisition	Blood loss	Decline in hemoglobin	Anemia	Transfusion
Small volume tubes	1; insufficient	2; insufficient	2; insufficient	1; suggestive	2; insufficient
Closed blood sampling devices	0; not applicable	4; moderate	7; insufficient	0; not applicable	4; insufficient
Point of care testing	0; not applicable	3; insufficient	0; not applicable	0; not applicable	2; insufficient
Bundled interventions	0; not applicable	4; suggestive	3; insufficient	0; not applicable	3; inconsistent
Education	2; insufficient	1; insufficient	1; insufficient	0; not applicable	1; insufficient

## Discussion

The large number of hospital ICU patients who develop anemia, and the contribution of blood taken for diagnostic testing to its development, is a longstanding concern [42–44]. Patients who develop anemia have poorer outcomes and higher risk of mortality, whether or not the patients are transfused [4, 44].

In this review, we examined the impact of interventions to reduce blood loss from diagnostic testing on the volume of blood lost, decline in hemoglobin, incidence of anemia, and transfusion rate. We found moderate, consistent evidence that blood conservation devices that return blood to the patient from flushing of venous or arterial lines or from in-line testing reduce the volume of blood loss by approximately 25%; relative reduction in blood loss was the same in NICU and adult ICU patients. Bundled interventions that included such devices reduced blood loss by a similar amount.

The evidence regarding the impact of blood conservation systems on the decline in hemoglobin over time or the incidence rate of anemia or transfusion was inconsistent. Most studies reported minimal impact on the decline in hemoglobin during the ICU stay, and two of four studies found increased transfusion among patients receiving the intervention. However, none of the studies used analytic methods that account for the interrelationship between hemoglobin levels and transfusions. Analyses that ignore this relationship or exclude transfused patients are likely to underestimate the effect of the intervention.

The evidence regarding the impact of routine use of small volume phlebotomy tubes was not as strong as that for closed blood sampling devices but generally supported their use. There was suggestive evidence that small volume tubes reduce the risk of anemia. The three studies that examined the effect on blood loss or hemoglobin decline found these outcomes were reduced when small volume tubes were used, but the evidence was insufficient for conclusions under our a priori criteria for assessing the body of evidence. All intervention bundles included small volume tubes. These bundles consistently reduced the volume of blood loss, but the effect on hemoglobin level was inconsistent.

The interventions examined in this review have been discussed multiple times [43, 45]. Institutions may not currently use blood conservation devices due to a number of concerns. One concern is that such devices may increase the risk of catheter-acquired infections. None of the studies of blood conservation devices in the review reported on this outcome. Blood conservation devices are also costly, and without evidence of impact on health outcomes, institutions may be reluctant to invest in the devices. The cost of the device may be offset by savings on the cost of blood for transfusions.

We found evidence on three additional interventions aimed at reducing blood loss for diagnostic testing and the associated risk of iatrogenic anemia and transfusions. These were point of care testing devices, policy changes and provider education aimed at reducing unnecessary testing, and bundles of interventions implemented together. The evidence was insufficient in supporting the effectiveness of these interventions.

The inability of our review to evaluate several commonly proposed interventions to reduce blood loss from diagnostic testing among critically ill patients illustrates the need for additional research and for improvements in research on this topic to improve both individual studies and future systematic reviews. Although we used recommended methodological practices to limit the risk of bias in our review, the available evidence required accommodations that may have introduced bias. Most notably, the included studies reported on different measurements of outcomes of interest. We identified the most clinically relevant measures among those commonly reported and attempted to convert reported results into those measures. This conversion sometimes required assumptions, such as using the average number of days of ICU admission and the average total blood loss to calculate blood loss per patient per day. In other cases, we were unable to convert the measures and had to consider similar but unequal measures, such as median loss per day rather than mean loss per day, as if they were equivalent. If the assumptions underlying these conversions and groupings were wrong, our findings may be biased in unpredictable ways. In addition, the body of evidence for any given outcome was small, limiting our ability to examine direct evidence across the causal chain.

Although the evidence limits our ability to assess the effectiveness of these interventions, this review highlights their potential and provides important guidance on future research. As mentioned above, a limitation of the existing evidence is how the studies that reported on the decline in hemoglobin accounted for transfused patients within the study population. The studies took one of three approaches, all of which potentially bias the study results: 1) They excluded any patient who was transfused; 2) They ignored the impact of the transfusion on the outcome; and 3) They reported on the outcome before the transfusion.

The interventions we discuss aim to reduce the amount of blood drawn or lost per blood drawn or per laboratory test. An alternative strategy would be to reduce the number of inappropriate laboratory tests ordered, thereby requiring fewer blood draws. Multiple studies and reviews have found that some routine laboratory tests ordered are of limited clinical value [15, 46–50]. Although interventions aimed at reducing blood loss by reducing laboratory testing were eligible for our review, we were unable to evaluate their effectiveness because the outcome measures reported for these studies [34, 41, 51] differed from each other and from the other studies in our review. A 2017 review of interventions aimed at reducing the use of routine testing found that the most effective approaches were education, clinician audit, and electronic medical record-enabled restrictive ordering [49].

Overall, our review highlights the potential these interventions have to reduce the amount of blood vulnerable patients lose during hospitalization, particularly in the ICU. Most studies and the clearest evidence were on single interventions. Multi-intervention approaches would be expected to have a greater impact, but the evidence was not sufficient to conclude this was true, or to compare the effectiveness of different intervention bundles.

The available evidence was limited by few studies assessing any given intervention-outcome pair, fair quality studies and different outcome measures, impeding our ability to judge the impact of the interventions. Future research might benefit from using more detailed analysis methods that account for clinical situations that may affect results such as the effect of transfusion on hemoglobin concentration. Agreement on standard measures for blood loss, decline in hemoglobin, and the incidence of anemia and transfusion would also maximize the value of future studies.

## Conclusion

The results of this systematic review support the use of blood conservation systems with arterial or venous catheters to eliminate blood waste when drawing blood for testing. Moderate, consistent evidence indicated that devices that return blood from testing or flushing lines to the patient reduce the volume of blood loss by approximately 25% among ICU patients, with a similar reduction for intervention bundles that included such devices (suggestive evidence). The evidence was insufficient to conclude the devices impacted hemoglobin levels or transfusion rates. Future research might benefit from using more detailed analysis methods that account for clinical situations that may affect results such as the effect of transfusion on hemoglobin concentration.

## Additional files

Additional file 1: Expert panel members. (DOCX 14 kb)

Additional file 2: Review protocol. (DOCX 57 kb)

Additional file 3: A-6 criteria for the strength of evidence ratings. (DOCX 13 kb)

Additional file 4: Characteristics of included studies. (DOCX 40 kb)

Additional file 5: Evidence tables. (DOCX 24 kb)

## Data Availability

Endnote databases of all retrieved citations and their inclusion or exclusion at each stage of review are available from the corresponding author.
