# Supplementary material for: Interventions to prevent iatrogenic anemia: a Laboratory Medicine Best Practices systematic review
Source: Crit Care. 2019 Aug 9;23:278. doi: 10.1186/s13054-019-2511-9 (PMC6688222; doi:10.1186/s13054-019-2511-9)
Supplement: Supplementary file 1 — Expert panel members. (DOCX 14 kb) [file 13054_2019_2511_MOESM1_ESM.docx]

**SDC1. LMBP Systematic Review on Preventing Phlebotomy-Related Anemia**

**Expert Panel Members**

**Julie Gayken MT (ASCP), CLC (AMT)**

Medical Laboratory Administrator and Consultant - Retired

**Sharon Geaghan, MD**

Associate Professor, Department of Pathology

Pediatrics Division

Stanford University School of Medicine

**James H. Nichols, PhD**

Professor of Pathology, Microbiology and Immunology

Medical Director of Clinical Chemistry and Point of Care Testing

Vanderbilt University School of Medicine

**Christine Litwin, MD**

Professor, Pathology and Laboratory Medicine

Medical Director, Clinical Immunology and Referral Testing

Medical University of South Carolina

**Patrick D. Carroll, MD, MPH**

Women and Newborns Program, Intermountain Healthcare

Division of Neonatology, Dixie Regional Medical Center

## Dennis Ernst

Director, Center for Phlebotomy Education, Inc

**Michael T McEvoy, PhD, NRP, RN, CCRN**

EMS Coordinator, Saratoga County, NY

**Adam C Salisbury, MD, MSc**

Department of Internal Medicine, Division of Cardiovascular Diseases

Saint Luke’s Mid-America Heart and Vascular Institute,

**Meera Viswanathan, Ph.D.**

Director, RTI-UNC Evidence-Based Practice Center
