# Supplementary material for: Interventions to prevent iatrogenic anemia: a Laboratory Medicine Best Practices systematic review
Source: Crit Care. 2019 Aug 9;23:278. doi: 10.1186/s13054-019-2511-9 (PMC6688222; doi:10.1186/s13054-019-2511-9)
Supplement: Supplementary file 2 — Review protocol. (DOCX 57 kb) [file 13054_2019_2511_MOESM2_ESM.docx]

**SDC2. Protocol**

**Interventions to Reduce Blood Loss from Diagnostic Testing and Prevent Iatrogenic Anemia**

A Laboratory Medicine Best Practices Review

**Table of Contents**

[**Background and Objectives** **3**](#_Toc441662974)

[**Analytic Framework for the Review** 3](#_Toc441662975)

[Components 4](#_Toc441662976)

[Key Questions 5](#_Toc441662977)

[**Methods** **5**](#_Toc441662978)

[Inclusion and exclusion criteria 5](#_Toc441662979)

[Data Abstraction and Data Management 9](#_Toc441662980)

[Individual Study Quality Appraisal 10](#_Toc441662981)

[Data Synthesis 10](#_Toc441662982)

[**Final Report and Draft Manuscript** **10**](#_Toc441662983)

[**References** **11**](#_Toc441662984)

# Background and Objectives

# Anemia and resulting transfusion of allogenic red blood cells in common in critically ill patients. Studies suggest that the proportion of patients who develop anemia by the third day after admission into an ICU is as high as 90% [[1](#_ENREF_1)]. Several patient–related factors such as reduced red blood cell (RBC) life span, a decrease in the production of erythropoietin and a blunted bone-marrow response, contribute to the development of anemia in these patients [[2](#_ENREF_2)]. However, repeated phlebotomy is a significant factor that contributes significantly to anemia in this population [[2](#_ENREF_2)]. Patients in ICUs often have central venous or arterial catheters. This makes the collection of blood more convenient and the easy access to blood exacerbates the risk for iatrogenic anemia (IA) given that many ICU’s have poor methods of monitoring the volume of blood drawn from a patient [[3](#_ENREF_3)].

The fact that transfusion has not been shown to improve survival in this patient population [[2](#_ENREF_2)] combined with the many risks associated with transfusion, e.g., infection, vascular overload, lung injury, sensitization, and transfusion reactions [[4](#_ENREF_4)] provides the impetus to design and test interventions that could reduce the risk of IA in this population.

One effective strategy to minimize the risks of transfusion in this setting focuses on adopting strict and clear transfusion guidelines, and several evidence-based guidelines already exist.

Another strategy that involves the laboratory is to address the causes of iatrogenic anemia and thus minimize the risks of severe anemia and the need for transfusion. Although a narrative review recently summarized strategies to prevent iatrogenic anemia [[3](#_ENREF_3)], the review was not directed at laboratory professionals, and we have not identified any systematic reviews on this topic. Strategies to minimize phlebotomy-related blood loss include

- Decision support guidance to reduce test ordering. There are dozens of papers on lab utilization management, although none we found were focused on anemia prevention in hospitalized patients.
- Non-invasive diagnostic tools that obviate the need for phlebotomy, and point-of-care tests that might reduce phlebotomy requirements. There are dozens of papers on non-invasive tests for monitoring oxygenation and RBC delivery (anemia), although these typically focus on efficacy in replacing invasive testing, not directly on anemia prevention.
- Small volume blood tubes. There is at least one high quality prospective study demonstrating that the use of small volume tubes reduces iatrogenic blood loss [[5](#_ENREF_5)].
- Devices and techniques that minimize blood loss during phlebotomy or analyte measurement. A narrative review of studies through 2011 identified 9 prospective trials of devices and concluded that these devices reduce iatrogenic blood loss and transfusion requirements [[3](#_ENREF_3)].
- Bundles that combine these strategies. Riessen et al found that a blood-conservation bundle decreased blood loss from 43 to 15 ml/day in ICU patients and reduced transfusion requirements [[6](#_ENREF_6)]. Similar findings were found by Tridente et al. (2015) [[7](#_ENREF_7)].
- Point of Care Testing. Requires less volume of blood & provides immediate feedback [[3](#_ENREF_3)]

This systematic review will identify and review publications that assess the impact of the interventions above on reducing blood loss and reducing the risk for IA among hospitalized patients, especially those in ICUs and NICUs.

# Analytic Framework for the Review

### Components

**Quality Issue**: Patients develop iatrogenic anemia and may require transfusion because of blood drawn for laboratory testing. Interventions to reduce the amount of blood drawn can reduce iatrogenic anemia and transfusions.

**Interventions:** Non-invasive diagnostic procedures; small volume blood tubes; devices and techniques that minimize blood loss or combination of this techniques; point of care (POC) testing, decision support or other strategies to reduce test ordering [scope of this review limited to studies that explicitly investigate test utilization management as an intervention to reduce blood loss from phlebotomy].

**Expected Intermediate Outcomes.** Reduced volume of blood drawn

**Expected Health Outcomes.** Reductions in decline in hemoglobin levels during admission, incidence of iatrogenic anemia, and transfusions

**Potential Harm.** Patient does not receive appropriate testing, resulting in compromised care.

**Figure 1 Analytic Framework Model**

**Quality Issue**

Patients develop iatrogenic anemia because of blood drawn for laboratory testing.

**Potential for Improvement**

Reduction in blood drawn, incidence of iatrogenic anemia

**Population**

Hospital In-patients; patients in critical care.

**Interventions**

Non-invasive diagnostic tests;

devices and techniques to minimize blood loss; POC testing;

Test utilization CDS specifically to reduce blood loss.

**Intermediate Outcomes**

Reduced blood loss from phlebotomy.

**Health Outcomes**

Reduced decline in Hemoglobin levels

Reduced iatrogenic anemia

Reduces need for transfusion

.

**Harms**

Inadequate blood for testing, need for additional blood draws;

Did not receive appropriate tests

###

### Research Question

What interventions reduce the volume of blood loss in hospitalized patients?

**Health Outcomes**

Does the intervention reduce the decline in hemoglobin levels during admission, the incidence of iatrogenic anemia, or the need for transfusion?

**Harms**

Does the intervention lead to inadequate blood for testing, a need for additional blood draws, or patients not receiving appropriate testing, resulting in compromised care?

# Methods

## Inclusion and exclusion criteria

**Table 1. PICOTS: Inclusion and exclusion criteria**

|  | Inclusion and Exclusion Criteria |
| --- | --- |
| **POPULATION** | Include: Hospital inpatients or patients with an overnight hospital stay of any age. Populations of special interest: patients in ICUs, newborns, coronary patients, ventilated patients, patients on renal replacement therapy, patients admitted for a bleeding problem, patients with high APACHE II scores. |
| **INTERVENTIONS** | Include: Devices and techniques to reduce blood loss, non-invasive diagnostic tests, POC testing, use of alternate specimen types, CDS to reduce unnecessary testing with the goal of reducing blood loss.  Exclude: Transfusion reduction or test utilization interventions not aimed at reducing blood loss or iatrogenic anemia. |
| **COMPARATORS** | Patients who did not receive the intervention. May be patients in other units of the same level of care, patients admitted and treated prior to onset of intervention, or patients in other facilities.  Internal controls: Patients treated with and without the intervention. |
| **OUTCOMES** | Volume of blood drawn; total blood lost to phlebotomy.  Incidence of iatrogenic anemia, change in hemoglobin from admission.  Blood transfusions |
| **HARMS** | Failure to receive appropriate tests |
| **Timeframe/ Duration** | Blood volume: 1980 or later. Transfusions or anemia: 1990 or later |
| **Study Designs** | Include: Randomized clinical trials, retrospective or prospective cohort studies, and pre-post studies, and case-control studies  Exclude: Studies with no comparator, including case series and case reports. |
| **Settings** | Hospital inpatient wards and critical care units |

**Search Strategies**

RTI will conduct focused searches of PubMed, Embase, Cochrane, Web of Science, Psychinfo, and CINAHL. An experienced research librarian will use a predefined list of search terms and medical subject headings (MeSH), when applicable. An example search is shown in Table 2. We will limit the search to articles published from 1980 to the present. Data on transfusions or anemia will only be used from studies published since 1990. We will also review the reference lists of pertinent review articles and all studies meeting our inclusion criteria and add any previously unidentified relevant papers.

RTI will work with the expert panel to identify sources of unpublished data, such as internal QA data from hospitals or data from completed but unpublished studies. We will include studies that meet our inclusion criteria and contain enough information on the research methods used for the risk of bias assessment.

Table 2. Illustrative Search Strategies (PubMed)

| **Search** | **Queries** | **Number of Citations** |
| --- | --- | --- |
|  | | |
| #1 | Blood Gas Analysis [MeSH] | 32,879 |
| #2 | Blood Specimen Collection [MeSH] | 13,928 |
| #3 | Diagnostic Tests [MeSH] | 8,938 |
| #4 | Phlebotomy [MeSH] | 5,174 |
| #5 | Unnecessary Procedures [MeSH] | 4,557 |
| #6 | Blood sampling [All Fields] | 11,332 |
| #7 | Haemoglobin [All Fields] | 30,075 |
| #8 | Laboratory [All Fields] | 1,646,568 |
| #9 | Laboratories [MeSH] | 25,699 |
| #10 | Clinical Laboratory Techniques [MeSH] | 2,337,248 |
| #11 | Phlebotomy [All Fields] | 7,044 |
| #12 | Blood Gas Analysis [MeSH] | 178,810 |
| #13 | Catheterization [MeSH] | 23,307 |
| #14 | Catheters [MeSH] | 9,654 |
| #15 | Point-of-Care Systems [MeSH] | 1,471 |
| #16 | Arterial Catheter [All Fields] | 995 |
| #17 | Arterial Line [All Fields] | 837 |
| #18 | Blood Conservation [All Fields] | 659 |
| #19 | Blood Management [All Fields] | 1,636 |
| #20 | Blood safety [All Fields] | 259 |
| #21 | Blood saving [All Fields] | 223 |
| #22 | Closed method [All Fields] | 2,201 |
| #23 | Closed system [All Fields] | 1,211 |
| #24 | Decision aid [All Fields] | 272 |
| #25 | Decision algorithm [All Fields] | 28,076 |
| #26 | Decision support [All Fields] | 121,615 |
| #27 | Feedback [All Fields] | 457,158 |
| #28 | Intervention [All Fields] | 79,399 |
| #29 | Noninvasive [All Fields] | 62,568 |
| #30 | Non-invasive [All Fields] | 136 |
| #31 | POC test [All Fields] | 862 |
| #32 | POCT [All Fields] | 591 |
| #33 | Point of Care Test [All Fields] | 2,225 |
| #34 | Point of Care Testing [All Fields] | 132,773 |
| #35 | Tube [All Fields] | 52,563 |
| #36 | Tubes [All Fields] | 146,667 |
| #37 | Anemia [MeSH] | 26,640 |
| #38 | Blood Volume [MeSH] | 46,628 |
| #39 | Hemoglobins/analysis [MeSH] | 64,629 |
| #40 | Iatrogenic Disease [MeSH] | 10,915 |
| #41 | Patient Safety [MeSH] | 7,293 |
| #42 | Anemic [All Fields] | 46,898 |
| #43 | Blood loss [All Fields] | 35,113 |
| #44 | Blood volume [All Fields] | 307,932 |
| #47 | Iatrogenic [All Fields] | 48,860 |
| #48 | Critical Care [MeSH] | 20,970 |
| #49 | Critical Illness [MeSH] | 238,591 |
| #50 | Hospitals [MeSH] | 16,102 |
| #51 | Inpatients [MeSH] | 65,732 |
| #52 | Intensive Care Units [MeSH] | 123,113 |
| #53 | Critical care [All Fields] | 34,114 |
| #54 | Critically ill [All Fields] | 1,011,323 |
| #55 | Hospital [text word] [All Fields] | 82,607 |
| #56 | Hospitalized [All Fields] | 41,624 |
| #57 | ICU [All Fields] | 89,479 |
| #58 | Inpatient [All Fields] | 86,479 |
| #59 | Intensive Care Unit [All Fields] | 12,583 |
| #60 | Neonatal Intensive Care Unit [All Fields] | 7,735 |
|  | Block 1 (Exposure) connected by OR | 3,795,356 |
|  | Block 2 (Intervention) connected by OR | 1,073,867 |
|  | Block 3 (Outcome) connected by OR | 1,364,549 |
|  | Block 4 (Population/Setting) connected by OR | 1,369,958 |
|  | Block 1 AND Block 2 AND Block 3 AND Block 4 | 3,845 |
|  | Published since 1980 | 3,452 |
|  | Published in English | 3,101 |
|  | **Without Duplicates** | 2,895 |

Two reviewers will independently review each retrieved citation and will use structured flow charts to determine whether it meets the inclusion criteria and to note the reason for exclusion. Disagreements between the reviewers on whether the article should be included will be resolved by a senior reviewer. The reviewers will meet to resolve any disagreement in the reason for exclusion.

## Data Abstraction and Data Management

RTI will create data abstraction tables with coding and abstraction instructions in an Excel worksheet. The coding will be consistent with that in the Laboratory Medicine Best Practices coding manual. The abstraction tables will cover 5 topics:

**Citation:** Title, authors, year published or submitted, journal or publication name, author affiliation, and source of funding.

**Study characteristics:** Study design, setting, time period, comparator, population, recruitment methods, sample size and response or participation rates, if applicable.

**Intervention:** Description, practice duration, completeness of implementation as designed, training, staff and resources required, and cost of implementation.

**Outcomes:** Outcome measures, measurement method, and recording method.

**Results:** Findings, effect size, statistical tests, p-values or confidence intervals

Staff abstractors and a senior abstractor will be assigned to each publication. The staff abstractors will perform the primary abstraction, and the senior abstractor will review and correct abstractions as appropriate.

## Individual Study Quality Appraisal

Each individual study will be assessed using the A-6 appraisal methodology. The methodology appraises each of four dimensions. Each study starts with a potential score of 10 and reviewers deduct points based on the presence and severity of problems in study quality. If any dimensions is rated zero, the study is excluded from the review. The four domains are:

**Study Characteristics.** Includes generalizability of study setting, representativeness of the study sample, potential biases due to study design, sample selection, comparison group, and study duration (3 points)

**Practice.** Includes the adequacy of the practice description and any potential bias from the practice implementation (2 points)

**Outcome Measure.** Includes the face validity of the outcome measure, and the expected accuracy, validity and potential for bias in the measurement and recording methods (2 points)

**Results and Findings**. Includes the sufficiency of the sample size, the appropriateness of the statistical analytic methods, and the potential for bias in the study implementation (3 points)

Drs. Whitehead and Meleth, from RTI, will review each study independently and assign the study a score. They will discuss any differences in score or conflict on inclusion and assign a consensus score. They will appraise the studies in EPPI4 using a quality assessment form customized for this review.

## Data Synthesis

The evidence for each key question will be summarized. RTI will qualitatively synthesize and interpret the evidence from included studies of all outcomes. They will aggregate data by meta-analysis if three or more studies are homogenous in design, report sufficient information for the analysis and are statistically homogenous. Clinical homogeneity will be assessed by the study’s PICOTS. Statistical heterogeneity of pooled analysis will be assessed using the chi-squared statistic and the I^2^ statistic (the proportion of variation in the study estimates due to heterogeneity.

# Final Report and Draft Manuscript

RTI will summarize the review findings and delineate recommendations for best practices guidelines with in evidence report and a draft manuscript, which will be distributed to the CDC COR and to each expert panel member for comment and review. The CDC COR will be responsible for final changes, for CDC clearance, for submitting the manuscript for publication, and will serve as corresponding author.

# References

1. Corwin, H.L., *Anemia and blood transfusion in the critically ill patient: role of erythropoietin.* Crit Care, 2004. **8 Suppl 2**: p. S42-4.

2. Page, C., A. Retter, and D. Wyncoll, *Blood conservation devices in critical care: a narrative review.* Ann Intensive Care, 2013. **3**: p. 14.

3. Jakacka, N., E. Snarski, and S. Mekuria, *Prevention of Iatrogenic Anemia in Critical and Neonatal Care.* Adv Clin Exp Med, 2016. **25**(1): p. 191-7.

4. Levi, M., *Twenty-five million liters of blood into the sewer.* J Thromb Haemost, 2014. **12**(10): p. 1592.

5. Sanchez-Giron, F. and F. Alvarez-Mora, *Reduction of blood loss from laboratory testing in hospitalized adult patients using small-volume (pediatric) tubes.* Arch Pathol Lab Med, 2008. **132**(12): p. 1916-9.

6. Riessen, R., et al., *A Simple "Blood-Saving Bundle" Reduces Diagnostic Blood Loss and the Transfusion Rate in Mechanically Ventilated Patients.* PLoS One, 2015. **10**(9): p. e0138879.

7. Tridente, A., et al., *The implementation of a blood conservation strategy in critical care on transfusion requirements.* Intensive Care Medicine Experimenta, 2015. **3(S1)**: p. A822.
