# Supplementary material for: Interventions to prevent iatrogenic anemia: a Laboratory Medicine Best Practices systematic review
Source: Crit Care. 2019 Aug 9;23:278. doi: 10.1186/s13054-019-2511-9 (PMC6688222; doi:10.1186/s13054-019-2511-9)
Supplement: Supplementary file 3 — A-6 criteria for the strength of evidence ratings. (DOCX 13 kb) [file 13054_2019_2511_MOESM3_ESM.docx]

| SDC 3. A-6 criteria for level of evidence ratings | | | | | |
| --- | --- | --- | --- | --- | --- |
| **Strength Ratings** | **# Studies** | **Effect Size Rating** |  | **Quality Rating** |  |
| High | ≥ 3 | Substantial | And | Good |  |
| Moderate | ≥ 2 | Substantial | And | Good |  |
|  | OR ≥ 3 | Moderate | And | Good |  |
| Suggestive | = 1 | Substantial | And | Good |  |
|  | OR ≥ 2 | Moderate | And | Good |  |
|  | OR ≥ 3 | Moderate | And | Fair |  |
| Insufficient* | Too few | Minimal |  | Fair |  |

***** Insufficient evidence does not rule out the potential value of a practice. Frequently, it indicates a need for additional evidence assessing the effect of the practice.
