# Supplementary material for: Interventions to prevent iatrogenic anemia: a Laboratory Medicine Best Practices systematic review
Source: Crit Care. 2019 Aug 9;23:278. doi: 10.1186/s13054-019-2511-9 (PMC6688222; doi:10.1186/s13054-019-2511-9)
Supplement: Supplementary file 4 — Characteristics of included studies. (DOCX 40 kb) [file 13054_2019_2511_MOESM4_ESM.docx]

**SDC 4. Characteristics of Included Studies**

| Author (Year) | Study Setting  Patient Population  Study Period | Study Design  Design Type  Sample Selection  Sample Size | Intervention  Comparator | Outcomes | Quality Appraisal Score |
| --- | --- | --- | --- | --- | --- |
| Dolman (2015)[1] | Academic medical center surgical intensive care unit (ICU)  Adult ICU patients  January 2011–October 2012 | Pre-post  Patients age ≥18 years admitted to surgical ICU for ≥ 48 hours  Total: 248, Intervention: 116, Comparator: 132 | I**ntervention:** Small volume or pediatric tubes  **Comparison:** Conventional volume tubes | Volume of blood loss for diagnostic testing Units of red blood cells transfused Incidence of anemia Deaths | 8 |
| Foulke (1989)[2] | Academic medical center medical ICU  Adult ICU patients  20 weeks (two 10-week periods) | Pre-post;  All patients in medical ICU  Total: 151. Intervention: 70. Comparator: 81 | **Intervention:** Total and 24-hr volume of blood drawn documented in chart  **Comparison:** Volume of blood drawn not recorded on chart | Volume of blood loss for diagnostic testing  Number of patients transfused  Deaths | 5 |
| Gleason (1992)[3] | Surgical ICU  Adult ICU patients  Not reported | Non-blinded randomized trial  All ICU patients admitted with arterial lines.  Patients randomized at admission.  Patients. Total: 68, Intervention: 31, Comparator: 37  Blood samples: Total: 1,657, Intervention: 784, Comparator: 873 | **Intervention:** Closed blood sampling device  **Comparison:** Open arterial line sampling | Volume of blood loss for diagnostic testing | 7 |
| Harber (2006)[4] | Referral hospital ICU  Adult ICU  August–November 2003 | Randomized trial, not blinded  Consecutively admitted ICU patients.  Total: 49, Intervention:24, Comparator: 25.  Intervention: Female: 12, Mean age (SD): 60 (15.7) years, Median (LQ-UQ) APACHE-II score: 17(13-22), Comparator: Female: 15, Mean age (SD): 54 (20.6) years, Median (LQ-UQ) APACHE-II score: 18 (12-24) | **Intervention:** Small volume tubes, conservative phlebotomy policy **Comparison:** Adult tubes, standard phlebotomy protocol | Volume of blood loss for diagnostic testing  Change in hemoglobin level Tests ordered  Number of patients transfused  Deaths | 6 |
| Hassan (2010)[5] | Children’s hospital  Pediatric general admission inpatients,  January 1997–December 2004 | Pre-post  Patients with para-pneumonic effusion  Total: 81, Intervention: 24, Simultaneous controls: 28, Historical controls: 29 | **Intervention:** Microsampling (capillary) tubes, reinfusion of blood drawn prior to obtaining sample; directive to minimize blood draws.  **Comparison:** No blood conservation orders (pediatric tubes, no reinfusion), Simultaneous: patients treated during study period without orders, historical: patients treated before intervention developed | Volume of blood loss for diagnostic testing  Change in hemoglobin level  Number of patients transfused | 5 |
| Henry (1986)[6] | Academic medical center surgical and cardiothoracic ICUs  Adult ICU patients  Not reported | Pre-post  Consecutive patients admitted to ICUs  Total: 20 | **Intervention:** Small volume tubes, educational intervention. / **Comparison:** Standard volume tubes, pre-intervention | Volume of blood loss for diagnostic testing | 4 |
| Kurniali (2014)[7] | Medical center  Adult general admission inpatients  January–October 2011 | Pre-post  Adult general admission inpatients for ≥ 48 hours. Excluded patients with medical conditions or medications that affect hemoglobin levels, central line placement, or ICU admission or anemia at admission  January 2011-October 2011.  Total: 479, Intervention: 203 (58% female), Comparator: 276 (63% female) | **Intervention:** Small volume or pediatric tubes  **Comparison:** Standard volume tubes | Change in hemoglobin level | 7 |
| MacIsaac (2003)[8] | Community hospital  Medical and surgical ICU  Adult ICU patients with arterial lines  May–August 2010 | Non-blinded randomized trial  Consecutive adult patients excluding cardio-thoracic surgical patients  Total: 160, Intervention: 80, Comparator: 80 | **Intervention:** Closed blood sampling device  **Comparison:** Standard arterial pressure line | Volume of blood loss for diagnostic testing  Change in hemoglobin level  Number of patients transfused | 9 |
| Madan (2005)[9] | Academic medical center  Newborn ICU patients who weighed <1000g  1998, 2002 | Pre-post  All eligible infants who survived ≥ 2 weeks  Total: 80, Intervention: 34, Comparator: 46  Intervention: Female: 22, White: 14, Non-Hispanic: 23  Comparator: Female: 22, White: 21, Non-Hispanic: 32 | **Intervention:** Point of care testing **Comparison:** Conventional laboratory methods | Number of transfusions,  Number of test requisitions |  |
| Mahdy (2009)[10] | Academic medical center  Ireland  Adult ICU  January–March 2006 | Non-blinded randomized trial  All patients with expected ICU staff of ≥3 days, blood samples collected in 1^st^ 3 days of admission  Intervention: 20 intervention, Control:19 | **Intervention:** Small volume tubes, blood conservation device.  **Comparison:** Standard arterial line and adult tubes. | Volume of blood loss for diagnostic testing | 6 |
| Mahieu (2012)[11] | Academic medical center  Belgium  Neonatal ICU  4 years (2 years pre, 2006-2007; and 2 years post intervention, 2008-2009) | Pre-post  All 1397 neonates admitted  Post-Intervention: 720, Pre-Intervention: 677 | **Intervention:** Point of care testing **Comparison:** Conventional laboratory testing | Volume of blood loss for diagnostic testing  transfusion rate | 7 |
| Martínez-Balzano (2017)[12] | Academic medical center  Adult ICU  Control: November– December 2014. Intervention: March– April 2015 | Pre-post  Medical record review  Not provided | **Intervention:** Educational intervention  **Comparison:** Before intervention. | Number of arterial blood gas test requisitions | 8 |
| Mukhopadhyay (2010)[13] | Academic medical center  Singapore  Adult ICU  Control: January–June 2008 Intervention: July 2008–March 2009 | Pre-post  All patients admitted to ICU, ≥18 yrs  Pre-Intervention: 80: Mean age 61.6 (± 18.3), 49 male, mean APACHE II score 18.6 (± 7)  Post-Intervention: 170. Mean age 60.5 (± 15.5), Male: 101, Mean APACHE II score 21.24 (± 7.8) | **Intervention:** Blood conservation device.  **Comparison:** Standard arterial line. | Change in hemoglobin level  Units of red blood cells transfused  Deaths | 6 |
| Mukhopadhyay (2011) [14] | Academic medical center  Singapore  Adult ICU  Control: January–June 2008 Intervention: July 2008–March 2009 | Pre-post  Subgroup analysis of previous study[13]  All patients admitted to ICU, ≥18 yrs and hemoglobin of ≥ 115 g/L at admission  Intervention: 78. Mean age 60.0 (+/- 17.3), Male: 51, APACHE II score 18.7 (+/-8.2);  Control: 50. Mean age 61.7 (+/-19.3), Male: 33 APACHE II score 18.0 (+/-7.6) | **Intervention:** Blood conservation device.  **Comparison:** Standard arterial line. | Change in hemoglobin level | 6 |
| Peruzzi (1993)[15] | Academic medical center ICU  Adult ICU  14 days. Dates not reported. | Blinded randomized trial  All eligible ICU  Total:100 patients  Control:50. Mean age: 57.6, Female: 22, Mean time in study: 3.6 days. Intervention:50 patients, mean age: 63.1, Female: 25. Mean time in study: 4.0 days | **Intervention:** Blood conservation device  **Comparison:** Standard arterial catheter tubing system. | Volume of blood loss for diagnostic testing  Change in hemoglobin level Number of patients transfused | 8 |
| Rezende (2010)[16] | Community hospital  Adult ICU inpatients, ventilated.  6 months, dates not given. | Non-blinded randomized trial  All eligible ICU patients  Total: 127  Control: 65 patients;  Intervention: 62 patients | **Intervention:** Blood conservation device  **Comparison:** Arterial or central venous catheter. | Change in hemoglobin level Number of patients transfused | 9 |
| Riessen (2015)[17] | Academic medical center  Germany  Adult ICU  2008, 2010 | Pre-post  All eligible ICU patients  Total: N=91  Control:41 patients. Mean age: 62.1 (SD +/-16.1). Female: 17.  Intervention: 50. Mean age: 63.3 (SD +/-14.7), Female: 16 | **Intervention:** Blood conservation device, small volume tubes, non-invasive testing  **Comparison:** Arterial lines, large volume tubes, invasive arterial blood gas analyses. | Volume of blood loss for diagnostic testing  Change in hemoglobin level  Number of patients transfused | 7 |
| Salem (1991)[18] | University teaching hospital and community hospital  Adult ICU  1 year, dates not reported. | Prospective observational study  Patient selection not described  Teaching hospital: 72 samples; Community hospital: 249 | **Intervention:** Point of care testing  **Comparison:** Conventional laboratory analysis | Volume of blood needed for point of care testing versus laboratory analysis | 6 |
| Sanchez-Giron (2008)[19] | Academic hospital Mexico  Adult general admission inpatients  6 weeks, dates not reported | Pre-post  All adult inpatients, emergency department patients excluded  Control: 227, 664 test requisitions. Median age: 50, Female: 102. Intervention: 246, 696 test requisitions. Median age: 52, Female: 116 | **Intervention:** Small volume tubes **Comparison:** Large volume tubes. | Volume of blood loss for diagnostic testing | 7 |
| Saxena (2003)[20] | Academic medical center  Adult surgical ICU  Comparator: February–April 2000  Intervention: May–September 2000 | Pre-post  All patients in the SICU  Number of patients not reported | **Intervention:** Revised SICU lab test panel, small volume tubes  **Comparison:** Large volume tubes, Previous SICU lab panel, routine noon and midnight tests. | Tests ordered  Volume of blood loss for diagnostic testing | 3 |
| Silver (1993)[21] | Academic medical center.  Adult medical ICU  7 days, dates not reported | Blinded randomized cross-over trial  All patients in ICU at start of study  Total: 31 Female: 14 | **Intervention:** Blood conservation device  **Comparison:** Patients using the conventional arterial line system. | Volume of blood loss for diagnostic testing  Number of phlebotomies  Blood volume discarded | 8 |
| Smoller (1989)[22] | Community hospital  Adult surgical ICU  Study period not reported | Pre-post  Intervention: Consecutively admitted patients  Control: Records from previous study Intervention: 41 patients  Control: 15 patients | **Intervention:** Small volume tubes  **Comparison:** Larger volume tubes | Volume of blood loss for diagnostic testing  Number of phlebotomies | 4 |
| Thorpe (2000)[23] | Teaching hospital  Adult ICU  February 1996– March 1997 | Blinded randomized trial  ICU patients with expected stay of ≥ 7 days, arterial line + 1 other catheter  Total: 102 Female: 35 | **Intervention:** Blood conservation device  **Comparison:** Conventional arterial line | Change in hemoglobin level  Units of red blood cells transfused | 9 |
| Widness (2005)[24] | 2 academic medical centers  Neonatal ICU  14 days, dates not reported | Non-blinded randomized trial  Assignment to control vs. intervention group stratified by birth weight  Total: 83 Intervention: 42, Comparison: 41 | **Intervention:** In-line monitor with automatic reinfusion of unused blood  **Comparison:** Conventional laboratory analysis | Volume of blood loss for diagnostic testing  Change in hemoglobin level | 9 |

**Included Articles**

1. Dolman HS, Evans K, Zimmerman LH, Lavery T, Baylor AE, Wilson RF, Tyburski JG: **Impact of minimizing diagnostic blood loss in the critically ill**. *Surgery* 2015, **158**:1083-1087; discussion 1087.

2. Foulke GE, Harlow DJ: **Effective measures for reducing blood loss from diagnostic laboratory tests in intensive care unit patients**. *Crit Care Med* 1989, **17**:1143-1145.

3. Gleason E, Grossman S, Campbell C: **Minimizing diagnostic blood loss in critically ill patients**. *Am J Crit Care* 1992, **1**:85-90.

4. Harber CR, Sosnowski KJ, Hegde RM: **Highly conservative phlebotomy in adult intensive care--a prospective randomized controlled trial**. *Anaesth Intensive Care* 2006, **34**:434-437.

5. Hassan NE, Winters J, Winterhalter K, Reischman D, El-Borai Y: **Effects of blood conservation on the incidence of anemia and transfusions in pediatric parapneumonic effusion: a hospitalist perspective**. *J Hosp Med* 2010, **5**:410-413.

6. Henry ML, Garner WL, Fabri PJ: **Iatrogenic anemia**. *Am J Surg* 1986, **151**:362-363.

7. Kurniali PC, Curry S, Brennan KW, Velletri K, Shaik M, Schwartz KA, McCormack E: **A retrospective study investigating the incidence and predisposing factors of hospital-acquired anemia**. *Anemia* 2014, **2014**:634582.

8. MacIsaac CM, Presneill JJ, Boyce CA, Byron KL, Cade JF: **The influence of a blood conserving device on anaemia in intensive care patients**. *Anaesth Intensive Care* 2003, **31**:653-657.

9. Madan A, Kumar R, Adams MM, Benitz WE, Geaghan SM, Widness JA: **Reduction in red blood cell transfusions using a bedside analyzer in extremely low birth weight infants**. *J Perinatol* 2005, **25**:21-25.

10. Mahdy S, Khan EI, Attia M, O'Brien BP, Seigne P: **Evaluation of a blood conservation strategy in the intensive care unit: a prospective, randomised study**. *Middle East J Anaesthesiol* 2009, **20**:219-223.

11. Mahieu L, Marien A, De D, Mahieu M, Mahieu H, Van H: **Implementation of a multi-parameter Point-of-Care-blood test analyzer reduces central laboratory testing and need for blood transfusions in very low birth weight infants**. *Clin Chim Acta* 2012, **413**:325-330.

12. Martínez-Balzano CD, Oliveira P, O'Rourke M, Hills L, Sosa AF: **An Educational Intervention Optimizes the Use of Arterial Blood Gas Determinations Across ICUs From Different Specialties: A Quality-Improvement Study**. *Chest* 2017, **151**:579-585.

13. Mukhopadhyay A, Yip HS, Prabhuswamy D, Chan YH, Phua J, Lim TK, Leong P: **The use of a blood conservation device to reduce red blood cell transfusion requirements: a before and after study**. *Crit Care* 2010, **14**:R7.

14. Mukhopadhyay A, See KC, Chan YH, Yip HS, Phua J: **Effect of a blood conservation device in patients with preserved admission haemoglobin in the intensive care unit**. *Anaesth Intensive Care* 2011, **39**:426-430.

15. Peruzzi WT, Parker MA, Lichtenthal PR, Cochranzull C, Toth B, Blake M: **A CLINICAL-EVALUATION OF A BLOOD CONSERVATION DEVICE IN MEDICAL INTENSIVE-CARE UNIT PATIENTS**. *Critical Care Medicine* 1993, **21**:501-506.

16. Rezende E, Ferez MA, Silva J, J M, Oliveira AM, Viana RA, Mendes CL, Toledo D, Ribeiro N, M C *et al*: **Closed system for blood sampling and transfusion in critically ill patients**. *Rev Bras Ter Intensiva* 2010, **22**:5-10.

17. Riessen R, Behmenburg M, Blumenstock G, Guenon D, Enkel S, Schafer R, Haap M: **A Simple "Blood-Saving Bundle" Reduces Diagnostic Blood Loss and the Transfusion Rate in Mechanically Ventilated Patients**. *PLoS One* 2015, **10**:e0138879.

18. Salem M, Chernow B, Burke R, Stacey JA, Slogoff M, Sood S: **Bedside diagnostic blood testing. Its accuracy, rapidity, and utility in blood conservation**. *Jama* 1991, **266**:382-389.

19. Sanchez-Giron F, Alvarez-Mora F: **Reduction of blood loss from laboratory testing in hospitalized adult patients using small-volume (pediatric) tubes**. *Arch Pathol Lab Med* 2008, **132**:1916-1919.

20. Saxena S, Belzberg H, Chogyoji M, Wilcox S, Shulman IA: **Reducing phlebotomy losses by streamlining laboratory test ordering in a Surgical Intensive Care Unit**. *Laboratory Medicine* 2003, **34**(10):728-732.

21. Silver MJ, Li YH, Gragg LA, Jubran F, Stoller JK: **Reduction of blood loss from diagnostic sampling in critically ill patients using a blood-conserving arterial line system**. *Chest* 1993, **104**:1711-1715.

22. Smoller BR, Kruskall MS, Horowitz GL: **Reducing adult phlebotomy blood loss with the use of pediatric-sized blood collection tubes**. *Am J Clin Pathol* 1989, **91**:701-703.

23. Thorpe S, Thomas AN: **The use of a blood conservation pressure transducer system in critically ill patients**. *Anaesthesia* 2000, **55**:27-31.

24. Widness JA, Madan A, Grindeanu LA, Zimmerman MB, Wong DK, Stevenson DK: **Reduction in red blood cell transfusions among preterm infants: results of a randomized trial with an in-line blood gas and chemistry monitor**. *Pediatrics* 2005, **115**:1299-1306.
