# Supplementary material for: Interventions to prevent iatrogenic anemia: a Laboratory Medicine Best Practices systematic review
Source: Crit Care. 2019 Aug 9;23:278. doi: 10.1186/s13054-019-2511-9 (PMC6688222; doi:10.1186/s13054-019-2511-9)
Supplement: Supplementary file 5 — Evidence tables. (DOCX 24 kb) [file 13054_2019_2511_MOESM5_ESM.docx]

**Supplemental Content 5. Evidence Tables**

| **SDC 5, Table 1: Evidence on Impact of Blood Conservation Devices** | | | | | | |
| --- | --- | --- | --- | --- | --- | --- |
| **Author (Year)** | **Outcome**  **(Converted units)** | **Sample Size** | **Comparison Cohort**  As Reported  Conversion Units | **Intervention Cohort**  As Reported  Conversion Units | **Mean Difference**  mL/patient/day | **Percent Change** |
| Gleason (1992){Gleason} | Volume of blood loss  (mL/patient/day) | Comparison: 873  Intervention: 784 | —^a^  69 | — ^a^  35 | 34 | 49% |
| MacIsaac (2003){MacIsaac} | Volume of blood loss  (mL/patient/day) | Comparison: 80  Intervention: 80 | Median: 133 mL,  3.1 days  42.9 | Median: 63 mL,  2.0 days  31.5 | 11.4 | 27% |
| MacIsaac (2003){MacIsaac} | Change in hemoglobin  (g/L/patient/day) | Comparison: 80  Intervention: 80 | Median: -4 g/L  1.3 | Median: -7 g/L  3.5 | -2.2 | -163% |
| MacIsaac (2003){MacIsaac} | Patients transfused  (number) | Comparison: 80  Intervention: 80 | 30 (38%) | 17 (21%) | 13 (17%) | 45% |
| Mukhopadhyay (2010) { Muk 2010} | Change in hemoglobin  (g/L/patient/day) | Comparison: 80  Intervention: 170 | —^a^  3.2 | —^a^  1.7 | 1.5 | 46% |
| Mukhopadhyay (2010) { Muk 2010} | Patients transfused  (number) | Comparison: 80  Intervention: 170 | 17 (21%) | 52 (31%) | -35 (-10%) | -48% |
| Mukhopadhyay (2011) { Muk 2011} | Change in hemoglobin  (g/L/patient/day) | Comparison: 50  Intervention: 78 | —^a^  4.9 | —^a^  4.6 | 0.3 | 6% |
| Peruzzi (1993) {Peruzzi} | Volume of blood loss  (mL/patient/day) | Comparison: 50  Intervention: 50 | Blood loss: 320.8, days: 3.6  89.1 | Blood loss: 260.3, days: 4.0  65.1 | 24.0 | 27% |
| Peruzzi (1993) {Peruzzi} | Hemoglobin reduction  (g/L/patient/day) | Comparison: 50  Intervention: 50 | 3.7^c^ g/dL, 3.6 days  3.9 | 1.0^c^ g/dL, 4.0 days  2.5 | 1.4 | 36% |
| Peruzzi (1993) {Peruzzi} | Transfusions | Comparison: 50  Intervention: 50 | 0.6 per patient  —^b^ | 0.7 per patient  —^b^ | -0.1 per patient | -17% |
| Rezende (2010){Rezende} | Hemoglobin reduction  (g/L/patient/day) | Comparison: 65  Intervention: 62 | 1.4 mg/dL, 13.1 days  1.0 | 0.7 mg/dL, 14.1 days  0.5 | 0.5 | 48% |
| Silver (1993){Silver} | Volume of blood discarded  (mL/patient/day) | Total: 31 | NR | NR | 22.4 mL/day  0.7 | NC |
| Thorpe (2000){Thorpe} | Hemoglobin reduction  (g/L/patient/day) | Comparison: 52  Intervention: 48 | 0.7^c^ | -1.3^c^ | -0.7 | -100% |
| Widness (2005){Widness} | Volume of blood loss (mL/kg/patient/day)^d^ | Comparison: 42  Intervention: 41 | —^a^  4.7^c^ | —^a^  3.1^c^ | 1.6 | 24% |
| Widness (2005){Widness} | Transfusion (mL/kg of red blood cells per infant) | Comparison: 42  Intervention: 41 | 46 | 38 | 8 | 17% |
| ^a^ Original report in conversion units, ^b^ Could not convert, ^c^read from graph, ^d^ standard unit for pediatric patients  NR, Not reported, NC, Could not calculate | | | | | | |

| **SDC 5, Table 2: Evidence on Impact of Small Volume Tubes** | | | | | |  |
| --- | --- | --- | --- | --- | --- | --- |
| **Author (Year)** | **Outcome** | **Sample Size** | **Comparison Cohort**  **As Reported**  **Converted** | **Intervention Cohort**  **As Reported**  **Converted** | **Mean Difference (Converted)** | **Relative Effect (Converted)** |
| Dolman (2015){Dolman} | Incidence of Anemia  (percent of cohort) | Comparison: 132  Intervention: 116 | —^a^  22.0% | —^a^  10.3% | 11.6% | 47% |
| Dolman (2015) {Dolman} | Volume of blood loss  (mL/patient/day) | Comparison: 132  Intervention: 116 | —^a^  31.7 | —^a^  22.5 | 9.2 | 29% |
| Harber (2006){Harber} | Volume of blood loss  (mL/patient/day) | Comparison: 25  Intervention: 24 | Median: 40  37.8 | Median: 8.0  8.3 | 29.5 | 78% |
| Harber (2006){Harber} | Hemoglobin reduction  (g/L/patient/day) | Comparison: 25  Intervention: 24 | 2.0 g/dL, 3 days  6.7 | 1.3 g/dL, 3 days  4.3 | 2.3 | 35% |
| Kurniali (2014){Kurniali} | Hemoglobin reduction  (g/L/patient/day) | Comparison: 276  Intervention: 203 | 1.4 g/dL, days NR  —^b^ | 1.3 g/dL, days NR  —^b^ | Adjusted^d^: -0.2  NC | NC |
| Sanchez-Giron (Total) (2008) {Sanchez} | Volume of blood loss (mL/patient/day) | Comparison: 227  Intervention: 246 | Median: 13.5 mL  1.0 | Median: 3.7 nL  0.3 | 0.7 | 73% |
| Sanchez-Giron (ICU) (2008) {Sanchez} | Volume of blood loss (mL/patient/day) | Comparison: 227  Intervention: 246 | Median: 19.9 mL  1.4 | Median: 5.1  0.4 | 1.1 | 74% |
| Smoller (1989) {Smoller} | Volume of blood loss (mL/patient/day) | Comparison: 15  Intervention: 41 | —^a^  55.6 | —^a^  32.2 | 23.4 | 42% |
| ^a^ Original report in conversion units, ^b^ Could not convert, ^c^ read from graph, ^d^ adjusted by multilinear regression for age, sex, race, ethnicity, BMI, length of stay and comorbities.  NR, Not reported, NC, Could not calculate | | | | | | |

| **SDC 5, Table 3: Evidence on Impact of Bundled Interventions** | | | | | | | |  |
| --- | --- | --- | --- | --- | --- | --- | --- | --- |
| **Author (Year)** | **Intervention Components** | **Outcome** | **Sample Size** | **Comparison Cohort**  **As Reported**  **Converted** | | **Intervention Cohort**  **As Reported**  **Converted** | **Mean Difference (Converted)** | **Relative Effect (Converted)** |
| Hassan (2010){Hassan} | Microsampling tubes, reinfusion of blood drawn prior to obtaining sample; directive to minimize blood draws. | Volume of blood loss  (mL/kg/patient/day) | Intervention: 24  Comparison (simultaneous controls): 28 | —^a^  0.14 | —^a^  0.08 | | 0.06 | 43% |
| Hassan (2010) {Hassan} | Microsampling tubes, reinfusion of blood drawn prior to obtaining sample; directive to minimize blood draws. | Hemoglobin level  (g/L/patient/day) | Intervention: 24 Comparison (simultaneous controls): 28 | 2.1 gm/dL  1.6 | 1.7 gm/dL  1.7 | | -0.1 | -6% |
| Hassan (2010) {Hassan} | Microsampling tubes, reinfusion of blood drawn prior to obtaining sample; directive to minimize blood draws. | Number patients transfused | Intervention: 24 Comparison (simultaneous controls): 28 | —^a^  5 (18%) | —^a^  2 (8%) | | 3 (10%) | 44% |
| Henry (1986){Henry} | Small volume tubes Education | Volume of blood loss  (mL/patient/day) | Comparison: 20  Intervention: 20 | —^a^  Cardio ICU: 377  Surgical ICU: 240 | —^a^  196  150 | | 181  90 | 48%  38% |
| Mahdy (2009){Mahdy} | Small volume tubes Blood conservation device | Volume of blood loss  (mL/patient/day) | Comparison: 19  Intervention: 20 | 45.4 mL, 3 days  15.0 | 15.2 ml, 3 days  5.1 | | 10.0 | 66% |
| Mahdy (2009){Mahdy} | Small volume tubes Blood conservation device | Hemoglobin level  (g/L/patient/day) | Comparison: 19  Intervention: 20 | 1.3 (units NR)  —^b^ | 0.79 (units NR)  —^b^ | | NC | 65% |
| Riessen (2015){Riessen} | Small volume tubes  Blood conservation device  Non-invasive testing | Volume of blood loss  (mL/patient/day) | Comparison: 41  Intervention: 50 | —^a^  43.3 | —^a^  15.0 | | 28.3 | 65% |
| Riessen (2015){Riessen} | Small volume tubes  Blood conservation device  Non-invasive testing | Hemoglobin level  (g/L/patient/day) | Comparison: 41  Intervention: 50 | 2.5 g/dL, LOS NR  —^b^ | 3.4 g/dL, LOS NR  —^b^ | |  | -36% |
| Riessen (2015) {Riessen} | Small volume tubes  Blood conservation device  Non-invasive testing | Number patients transfused | Comparison: 41  Intervention: 50 | 13 (32%) | 4 (8%) | | 9 (24%) | 75% |
| Saxena (2003){Saxena} | Revised lab test panel  Small volume tubes | Volume of blood loss (scheduled draws only)  (mL/patient/day) | NR | —^a^  35^c^ | —^a^  20 | | 15 | 43% |
| ^a^ Original report in conversion units, ^b^ Could not convert, ^c^ Based on midpoint of range of midnight scheduled draws.  NR, Not reported, NC, Could not calculate, LOS, length of stay | | | | | | | | |

| **SDC 5, Table 4: Evidence on Impact of Other Interventions** | | | | | | |  |
| --- | --- | --- | --- | --- | --- | --- | --- |
| **Author (Year)** | **Intervention** | **Outcome** | **Sample Size** | **Comparison Cohort**  **As Reported**  **Converted** | **Intervention Cohort**  **As Reported**  **Converted** | **Mean Difference (Converted)** | **Relative Effect (Converted)** |
| Foulke (1989) | Documentation of blood drawn | Volume of blood loss  (mL/patient/day) | Intervention: 70 Comparison: 81 | —^a^  62.6 | —^a^  37.8 | 24.8 | 40% |
| Foulke(1989) | Documentation of blood drawn | Number of patients transfused | Intervention: 70 Comparator: 81 | 8 (10%) | 1 (1%) | 7 (9%) | 90% |
| Martínez-Balzano (2017) | Educational intervention | Number of arterial blood gas test requisitions per patient per ventilated days | NR | 2.3^c^ | 1.3^c^ | 1.0 | 43% |
| Madan (2005) | Point of care testing | Number of transfusion per patient | Intervention: 34, Comparator: 46 | 5.7  —^b^ | 3.1  —^b^ | 2.6 | 46% |
| Mahieu (2012) | Point of care testing | Volume of blood loss  (mL/patient/day) | Intervention: 720 Comparison: 677 | 6,056 mL  —^b^ | 4,913 mL  —^b^ | 1,639 | 27% |
| Mahieu (2012) | Point of care testing | Number of patients transfused by birthweight | Intervention: 720 Comparison: 677 | <1500g: 50%  1500-2500g: 9%  >2500g: 6 | 39%  9%  10% | 11%  0.2%  -4% | 22%  2%  -67% |
| Salem (1991) | Point of care testing | Volume of blood loss  (mL/patient/day) | Total: 321 | All tests: NR | NR | 1,614 mL  —^b^ | NC |
| ^a^ Original report in conversion units, ^b^ Could not convert, ^c^ Read from graph.  NR, Not reported, NC, Could not calculate, LOS, length of stay | | | | | | | |
